# Optimization Design of Magnetic Isolation Ring Position in AC Solenoid Valves for Dynamic Response Performances

**DOI:** 10.3390/mi13071065

**Published:** 2022-07-02

**Authors:** Jiang Guo, Linguang Li, Pu Qin, Jinghao Wang, Chao Ni, Xu Zhu, Dingyao Lu, Jiwu Tang

**Affiliations:** 1 Key Laboratory for Precision and Non-Traditional Machining Technology of Ministry of Education, Dalian University of Technology, Dalian 116024, China; guojiang@dlut.edu.cn (J.G.); lilinguang@mail.dlut.edu.cn (L.L.); qinpu@mail.dlut.edu.cn (P.Q.); 534657972@mail.dlut.cn (J.W.); nichao@mail.dlut.edu.cn (C.N.); 2 Ningbo Institute of Dalian University of Technology, Ningbo 315000, China; zhux_nbi@dlut.edu.cn; 3 Jiayin Electromechanical Technology Co., Ltd., Ningbo 315400, China; ldy@jiayin.biz; 4 College of Applied Technology, Dalian Ocean University, Dalian 116300, China

**Keywords:** optimization design, magnetic isolation ring, dynamic response characteristics, simulation

## Abstract

Dynamic response characteristics of solenoid valves directly determined their performances. Among numerous parameters, the influence of magnetic isolation ring (MIR) on solenoid valve performance is crucial. Previous optimization studies have not conducted a systematic exploration and analysis of MIR. In this paper, a model of an AC solenoid valve considering the position of the MIR is proposed, and the model’s accuracy was verified by simulation and experiments. The electromagnetic force, response time, and magnetic field distribution at different positions of the MIR were analyzed, and the effect of the position of MIR on dynamic response characteristics of the solenoid valve was clarified. The results show that the MIR affects the dynamic response characteristics of the solenoid valve by changing the magnetic circuit. With the positive translation of the position of the MIR along the Z-axis, the electromagnetic force first increases and then decreases, and the response time first decreases and then increases. The position range of MIR with excellent dynamic response performance was obtained from the comprehensive consideration of response time and electromagnetic force. Finally, the optimization design for the dynamic response performance of the solenoid valves is realized.

## 1. Introduction

Solenoid valves are electromagnetic control elements used to control the flow, velocity, and direction of various media. They have many significant advantages, such as simple structure, low cost, rapid response, and high reliability. Therefore, solenoid valves play an essential role in aerospace, oil and gas transportation, industrial refrigeration, and other fields [1,2,3,4]. With the development of the industrial level, the requirements of miniaturization and high performance of solenoid valves in production are more urgent. Dynamic response characteristics of solenoid valves (i.e., response time, electromagnetic force, and magnetic induction intensity) are the most direct indicators to measure their operating performance, and it determines the comprehensive performance of the solenoid valve [5,6]. In order to obtain better solenoid valve performance, its structure needs to be optimized. The traditional design method is to determine the performance parameters of the valve through an empirical formula and analytical calculation. Then, the valve performance is verified through the prototype test. Finally, the design level is improved according to the feedback of the experimental results. This technical scheme based on theoretical calculation and experimental verification have the problems of a longer product development cycle, higher cost, and lower efficiency, which is gradually tricky to implement in meeting the needs of industrial development [7,8].

The finite element simulation technology is widely used in various optimization design links [9,10,11]. This technology is also combined with the optimized design of the solenoid valve. The optimization design method of solenoid valve based on finite element simulation is formed, which can overcome the limitations of traditional design. In recent years, researchers have done a great deal of simulation work for the optimization design of the structural parameters for the solenoid valve. Shukla et al. [12] verified the relationship between the external magnetic field strength and the movement of the moving iron core through simulation and experiments. Subic et al. [13] used the simulation method to explore reducing the solenoid valve’s structural size while maintaining the solenoid valve’s performance. Hung et al. [14] explored the influence of the solenoid spring stiffness, plunger mass and other structural parameters on the working characteristics. Grekhov et al. [15] found that the complete demagnetization of the iron core can achieve a faster response. Liu et al. [16] analyzed the core structure, coil structure, armature structure, and the effect of changes in the working air gap and drove current on the electromagnetic force of the solenoid valve. Hung et al. [17] found that the thickness of the solenoid valve cut-off sleeve, the cross-sectional shape of the coil, and the relative position of the coil and the plunger in the fuel injector have a significant influence on the electromagnetic force of the fuel injector. Yang et al. [18] found that the smaller the number of coils turns in the miniature digital valve, the more excellent the resistance and the faster the response of the electromagnet. Zhao et al. [19] studied the effect of eddy current on the dynamic response of high-speed solenoid valve of common rail injector and found that eddy current always hinders the change of magnetic field, which makes the dynamic response of high-speed solenoid valve worse. Slotting the yoke significantly reduces opening response time. Liao et al. [20] determined that the length of the casing, the length of the valve seat, and the height of the boss do not have a linear relationship with the electromagnetic force, but there is an optimal solution, and the study obtained the point with the largest electromagnetic force through simulation. At present, the research on the dynamic characteristics of solenoid valves mainly focuses on material optimization, external circuit optimization, and structure optimization. Among them, the main direction is the research on the coil structure and the shape of the armature in the structural part. 

The concept of high-performance manufacturing is widely recognized and applied in modern manufacturing [21]. Performance-oriented manufacturing is a top-down reverse manufacturing process. Among them, performance-oriented design is an important core of high-performance manufacturing. However, the current research on solenoid valves does not fully meet the optimization requirements for dynamic response performance. At the same time, the magnetic isolation ring (MIR) is a critical component that affects the dynamic response characteristics of the solenoid valve. According to theoretical analysis, the position of the MIR significantly impacts the solenoid valve’s response time and the armature’s pull-in force [22]. The research on its influence law is still not systematic and in-depth.

In the present study, we incorporated the idea of high-performance manufacturing into the optimized design of solenoid valves and emphasized the optimization design of AC solenoid valves for dynamic response performances. The position of the MIR, which was more critical and rarely mentioned in previous studies, was taken as the research object. A model of an AC solenoid valve considering the position of the MIR was proposed and established. In order to verify the simulation results, a series of innovative solenoid valve dynamic response characteristic measurement devices were developed. The influence law of the position of the MIR on its dynamic response characteristics was deeply explored and analyzed. The research results can provide important references for developing high-performance optimization of solenoid valves.

## 2. Modeling of the AC Solenoid Valve

**Figure 1:**
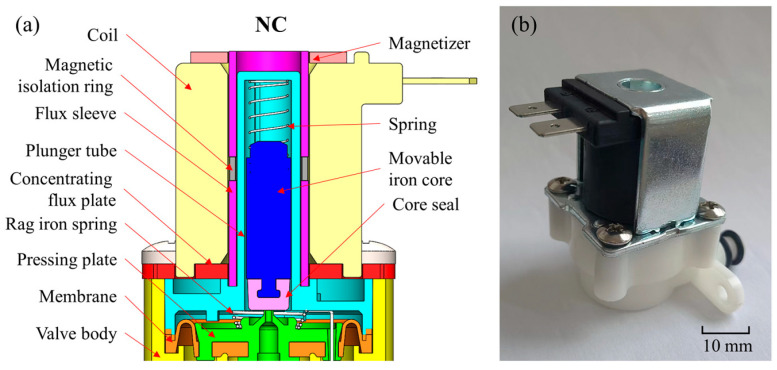
(**a**) Structural diagram and (**b**) optical image of the solenoid valve; the initial working air gap of the model is 12.16 mm, and the working stroke of the movable iron core is 5 mm.

The experimental research is oriented to the standard AC normally closed solenoid valve. The structural diagram and optical image of the solenoid valve is shown in Figure 1. The size of the solenoid valve is 60.8 mm (length) × 34.0 mm (width) × 53.8 mm (height). When the solenoid valve is not in operation, the coil is powered off, and the magnetic induction intensity in the air gap is zero. At this time, the movable iron core is not subjected to the electromagnetic force and moves downward to close the pilot valve port under the action of the spring force. The cavity pressure on the diaphragm increases, and the diaphragm is down under the action of spring force and differential pressure force to close the main valve port.

In the experiment, the AC solenoid valve with a voltage of 36V was selected as the simulation object. The voltage input is sinusoidal AC voltage. Because the form of current excitation will directly determine the magnitude, direction, and variation trend of the electromagnetic force, the accuracy of the established current excitation model has an essential impact on the final calculation results. In Maxwell’s default excitation form, only constant current excitation cannot restore the commutation behavior of sinusoidal alternating current in the excitation process. Therefore, this experiment uses the external circuit field to build a bridge rectifier circuit to construct the excitation source to simulate the actual working state of the solenoid valve more accurately. The mathematical circuit model of the electromagnetic coil can be expressed as

(1) $$V\left(t\right)=V_{0}+V_{a}\cdot e^{-Df\left(t-T_{d}\right)}\cdot \sin\left[2\cdot \pi \cdot IFreq\cdot \left(t-T_{d}\right)-Phase\right]$$

where *V*_0_ is the offset voltage in volts, *V_a_* is the peak amplitude in volts, *IFreq* is the signal frequency, *T_d_* is the delay time in seconds, Phase is the signal phase delay, and *Df* is the damping factor in 1/seconds. 

The material properties of the main structure are defined as Table 1. MIR needs to be defined as a solid conductor with a voltage source. Definition of properties can be expressed as

(2) $$\iint_{\Omega_{c}}\left(\sigma \frac{dA}{dt}+j_{t}\right)d\Omega =\iint_{\Omega_{C}}\;\frac{\sigma }{l}V_{b}d\Omega$$

where $\Omega_{c}$ is the width cross-section of the *n*th conductor, *V_b_* is the known voltage source between the two conductors, ***j_t_*** is the total current density to be solved for, *σ* is the conductivity, *l* is the thickness of the model, and ***A*** is the magnetic vector potential. 

**Figure 2:**
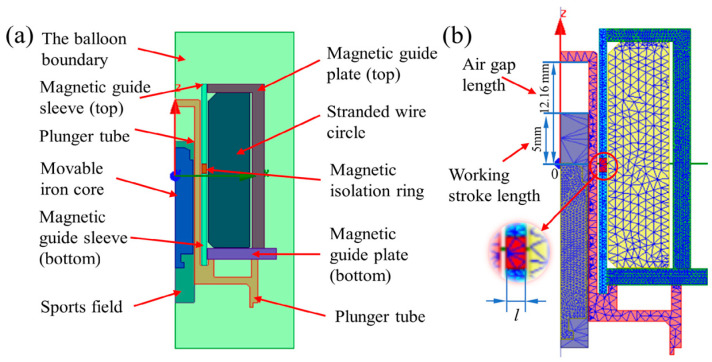
(**a**) Solution settings and (**b**) the meshing length of the magnetic conductive material is set to 0.5 mm, and the rest are set to 1.5 mm.

The motion domain is set as shown in Figure 2a. The motion direction of the movable iron core is set to be along the positive Z-axis. Motion types fall within the range of transient motions. The equation of motion for translational motion can be expressed as

(3) $$ma+\lambda v=F_{em}+F_{load}$$

where *m* is the mass of the object, a is the acceleration of the object, v is the velocity, $F_{em}$ is the computed electromagnetic force, $F_{load}$ is the external load force, and λ is the damping.

The electromagnetic force realizes the opening and closing behavior of the solenoid valve on the movable iron core overcoming the spring load force. Thus, the accurate definition of the spring load force directly determines the calculation results of the dynamic response characteristics of the solenoid valve. Since the spring load force change is not a monotonic linear relationship, the pwlx function defines the spring load force in this experiment. The Hooke coefficient is defined as 0.024 N/mm, the initial position is set to 0 mm, and the maximum displacement is set to 5 mm along the positive Z-axis, which is equal to the working stroke of the movable iron core. The iron core weight is set to 0.005 kg, and the damping coefficient is 1 × 10^−5^. The specific value is obtained by experimental measurement.

The established solenoid valve simulation model is a 2D model. The model is of solenoids and insulators with rotational symmetry axis. The coordinate system adopts the cylindrical coordinate system (*rφz*), the *z*-direction is the axial direction, and the *r* direction is the radial direction. Since any symmetry plane is the *rz* plane, there is no displacement in the *φ* direction. The electric or magnetic field in the calculation results must also be rotationally symmetric. Because ***F*** has a *ϕ*-component only, these operators are defined as

(4) $$\nabla \times F=\left(\frac{1}{r}\right)[-\frac{\hat{\partial }\left(rF_{\phi }\right)}{\partial z}\hat{r}+\frac{\partial \left(rF_{\phi }\right)}{\partial r}\hat{z}$$

where *F* is a scalar quantity, and ***F*** is a vector quantity.

Since the model is axisymmetric, the boundary condition of the solution domain is set to the balloon boundary. The balloon boundary simulates the outer region as near infinity. This boundary condition effectively isolates the model from other electrical current or magnetic fields sources.

The coil is set to add heat form, and the number of turns is set to 2700. A winding is defined as an external circuit with an initial voltage of 0. The external circuit setting form is shown in the figure. The internal division based on length is selected as the meshing form. The movable iron core and the magnetic conductor are our key observation objects for the magnetic ring. The meshing of the model is shown in Figure 2b. The free meshing method is adopted to mesh the solenoid valve. The meshing density of magnetically conductive material should be larger to improve the simulation accuracy, which is set to 0.5 mm. The meshing division of other non-magnetic materials, such as the solution domain, can reduce the accuracy to improve the simulation efficiency, which is set to the system calculation value of 1.5 mm.

In modeling of magnetic induction intensity, the excitation source, material permeability, coercive force of the permanent magnet, and motion parameters of fixed components in the model are considered core factors. The vectors have only one component in the Z-direction. The *v* needs to be set to 0 in the transient solution. Because the moving components have now been fixed to their own coordinate system, the partial time derivative becomes the total time derivative of ***A***. The time-dependent magnetic equation is expressed as

(5) $$\nabla \times v\nabla \times A=J_{s}-\sigma \frac{dA}{dt}-\sigma \nabla V+\nabla \times H_{C}$$

where *H_c_* is the coercivity of the permanent magnet, v is the velocity of the moving parts, ***A*** is the magnetic vector potential, ***V*** is the electric potential, *σ* is the reluctance, and ***J_s_*** is the current source density.

Then, the electromagnetic force is solved by using the Maxwell stress tensor, and the equation is expressed as

(6) $$T_{ij}\equiv \varepsilon_{0}\left(E_{i}E_{j}-\frac{1}{2}\delta_{ij}E^{2}\right)+\frac{1}{\mu_{0}}\left(B_{i}B_{j}-\frac{1}{2}\delta_{ij}B^{2}\right)$$

where ***T*** is the stress tensor, ***E*** is the electric field, ***B*** is the magnetic field, *ε*_0_ is the electric constant, *μ*_0_ is the magnetic constant, and *δ_ij_* is the Kronecker function. 

The force density flow is obtained by calculating the inner product of ***T*** and ∇. The equation is expressed as

(7) $${\left(\nabla \cdot \overset{↔}{T}\right)}_{j}=\varepsilon_{0}\left[\left(\nabla \cdot E\right)E_{j}+\left(E\cdot \nabla \right)E_{j}-\frac{1}{2}\nabla_{j}E^{2}]+\frac{1}{\mu_{0}}[\left(\nabla \cdot B\right)B_{j}+\left(B\cdot \nabla \right)B_{j}-\frac{1}{2}\nabla_{j}B^{2}\right]$$

Equation (8) integrates over the full space. The electromagnetic force calculation equation is expressed as

(8) $$F=\iint_{S}\overset{↔}{T}da$$

where ***F*** is the electromagnetic force on the moving iron core.

## 3. Design and Development of Measurement Platform

**Figure 3:**
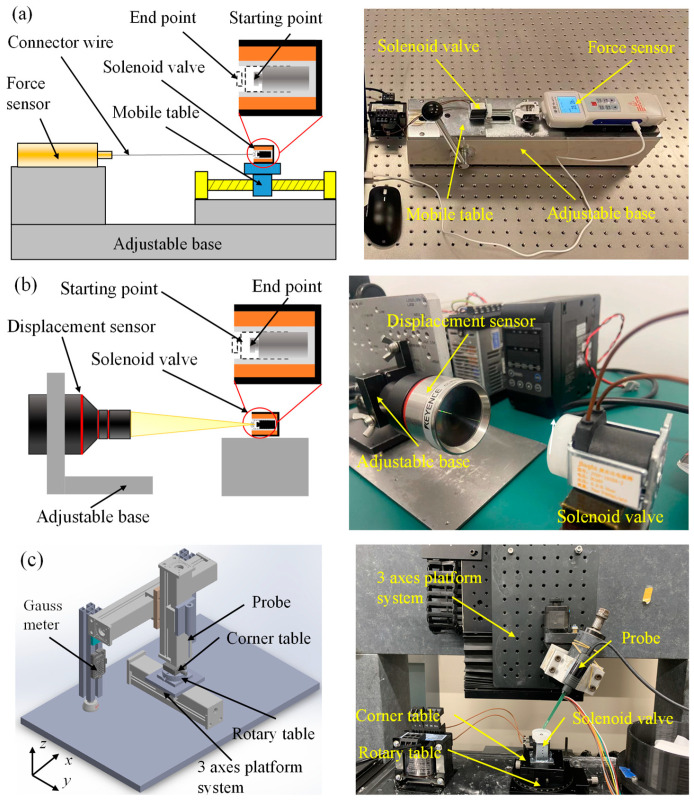
Experimental schematic diagram of measurement platform. (**a**) Electromagnetic force measurement with a measurement accuracy of 0.01 N, (**b**) response time measurement with a resolution of not less than 0.025 μm, and (**c**) magnetic field strength measurement with a measurement accuracy of 0.01 mT.

The leading indicators to measure dynamic response characteristics of solenoid valve include electromagnetic force, response time, and magnetic field strength. There are many solenoid valves with different functional types and structural dimensions in the industrial field. It is difficult for the existing detection devices to meet the current requirements for the universal measurement of solenoid valves. In order to meet the detection requirements of micro-miniature solenoid valves, a detection platform for the dynamic response characteristics of solenoid valves was designed and developed based on the principle of highly universal measurements. The platform is shown in Figure 3.

Figure 3a shows the schematic diagram of the electromagnetic force measurement platform. The electromagnetic force measurement is mainly based on the resistance strain gauge sensor. The strain effect reflects the electromagnetic force. It has the advantages of high resolution and tiny errors. It is suitable for static and fast alternating stress measurement. At the same time, the centering adjustment structure of the solenoid valve and the dynamometer are set to reduce the measurement error, which can realize the center alignment of the moving iron core and the dynamometer. The connector wire selects high-performance fibers with low elastic modulus to reduce the influence of the deformation of the connecting line on the measuring force. The moving iron core is connected with the digital display force-measuring instrument through the connector wire in the actual measurement. The electromagnetic force on the moving iron core is transmitted to the digital display force-measuring instrument through the thin wire during the pull-in process. Finally, data collection and processing will be carried out through a special electromagnetic force acquisition software. The electromagnetic force-measurement device designed can obtain the force curve of the moving iron core in the whole process of moving, and the measurement resolution reaches 0.01 N.

Currently, the measurement methods of response time mainly include the eddy current method, current-voltage analysis method, and displacement detection method, etc. [19,23]. Nevertheless, there is still a lack of equipment that is easy to implement and reliable in performance. Using the displacement detection method to realize the measurement of response time has high reliability by analyzing technically. The laser-based coaxial displacement sensor has the technical advantages of high sampling frequency and wide measurement range among the high-speed cameras and displacement sensors currently used in displacement measurement. Based on the preliminary design analysis and the design concept of high measurement accuracy and easy operation, the detection platform is settled to base on the coaxial laser displacement detection scheme. Figure 3b shows the response time detection platform designed and developed. The platform uses the Keyence CL-P070 laser color coaxial displacement sensor with good accuracy and an extensive measurement range. The displacement measurement range is ±10 mm, and the resolution is not less than 0.025 μm. It is also equipped with a self-aligning structure to reduce errors. The response time of the solenoid valve is measured by collecting the displacement-time curve between the moving iron core and the laser displacement sensor.

The magnetic field strength is mainly measured by using a Gauss meter. In measuring the magnetic field strength of the solenoid valve, it is difficult for the Gauss meter probe to accurately approach the measurement position since the pipeline where the moving iron core is located is relatively narrow, with the aperture usually narrower than 1 cm. For this reason, a rigid and precise measuring platform must be developed. In order to measure the magnetic field strength of the solenoid valve, a detection platform for the magnetic induction strength of the solenoid valve is developed, as shown in Figure 3c. The detection platform mainly has manual and automatic control functions. The motion accuracy is less than or equal to 0.05 mm. Furthermore, a C-axis precision turntable and an angle stage are designed on the worktable, which can flexibly and accurately adjust the position of the solenoid valve and expand the measurement range of the Gauss meter. The gaussmeter probe is used to perform fixed-point detection on the solenoid valve in the state of power in the actual measurement. The magnetic field strength of the measurement position is output through the measurement software.

## 4. Results and Discussion

### 4.1. Simulation Results

In order to better explore the influence of the position of the MIR on the electromagnetic force curve and response time, a single-term rectifier circuit in the simulation work was added based on the constant model. The internal circuit resistance was reduced. The influence law of the MIR’s position on the solenoid valve’s dynamic response characteristics is revealed more intuitively. As shown in Figure 2b, the red part is the MIR. The position of the MIR in Figure 2b is set as the zero-point *x*_0_ (*x_z_* = 0 mm), and the position of the MIR is adjusted along the direction of the Z-axis. The effects of different MIR positions on the solenoid valve’s response time *t* and the electromagnetic force are investigated by simulation.

The moving distance of the MIR relative to the initial position is defined as *x_z_*, the time from turning on the power supply to the moving iron core reaching the working position is defined as the response time *t*, and the electromagnetic force received by the moving iron core after the suction is defined as *f*. The relationship between the position of the MIR, the response time, and the electromagnetic force are shown in Table 2. With the positive change in the position of the MIR, the response time of the solenoid valves, the electromagnetic force, and the displacement of the MIR do not have a linear relationship. However, there is an optimal position interval that takes both into account. With the positive increase of *x_z_*, the electromagnetic force increases and then decreases, and the maximum value is 1.416 N. The response time gradually increases. The response time decreases first and then increases, and the minimum value is 13.0 ms.

When the position of the MIR is within 1.5 mm, the electromagnetic force when the armature is pulled in reaches the highest level, and then, the magnitude of the electromagnetic force begins to decrease gradually. When the position of the MIR is within −1.5 mm, the valve has the shortest response time. The MIR continued to be moved down, increasing the response time slowly.

### 4.2. Experimental Verification

In order to verify the accuracy of the simulation model, a model verification study was carried out using the existing AC solenoid valve models based on the measurement platforms mentioned in Section 3. The AC solenoid valve with the MIR located at 0.5 mm was measured. The measured resistance value of the solenoid valve coil is 1378 Ω. The circuit in the experiment uses a simplified circuit without diodes. According to the requirements of the experimental conditions, an additional simulation of the solenoid valve selected in the experiment is carried out to verify the validity of the simulation model.

**Figure 4:**
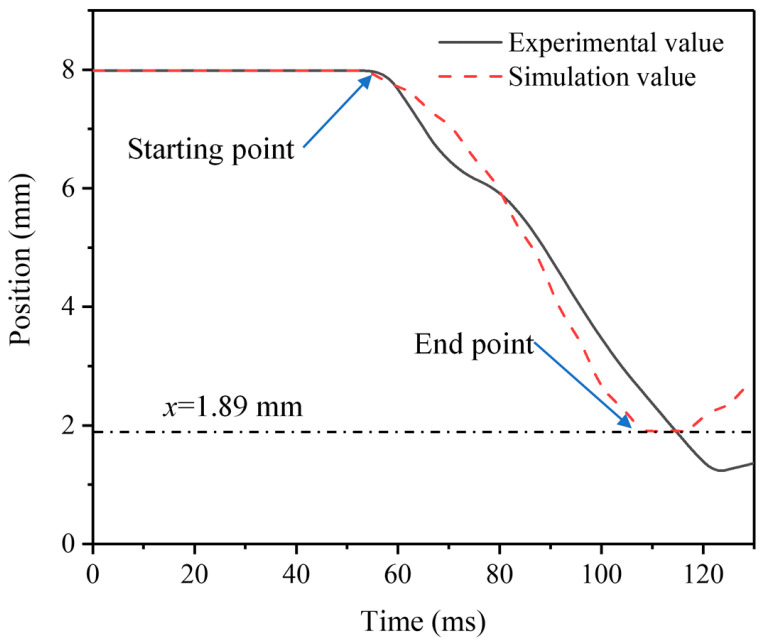
Measurement results of response time.

The measurement results show that when the moving iron core stroke reaches the end position, the electromagnetic force is 0.37 N, and the error compared with the simulation results is only 14.10%. The simulation results are in good agreement with the experimental results, and the simulation error is kept within 10%. The experimental results of the response time are shown in Figure 4. The test results record the displacement process of the moving iron core from the initial position to the end position as well as the start time and end time. The calculated response time is 63 ms. Compared with the simulation result of 59 ms, the error is only 6.35%. The magnetic induction intensity is measured on the top right corner of the magnetic guide plate, and the measurement result is 1.64 mT. The simulation results show that the magnetic induction intensity is 1.52 mT, and the simulation error is only 7.31%.

The dynamic response performances of the AC solenoid valve were measured by using the corresponding measurement platforms. The experimental results are in good agreement with the simulation results. The simulation error is generally stable at about 10%, which verifies the accuracy of the simulation model. On the other hand, the experimental verification further proves that the developed dynamic response characteristic measurement platforms have the excellent capability.

### 4.3. Effect of MIR Position on Electromagnetic Force and Response Time

In order to clarify the dynamic response performance of the solenoid valves, it is necessary to explore the dynamic change process of the electromagnetic force and the position of the movable iron core during the pull-in process of the movable iron core. Therefore, the simulation method is used to analyze the dynamic response process of the solenoid valves.

**Figure 5:**
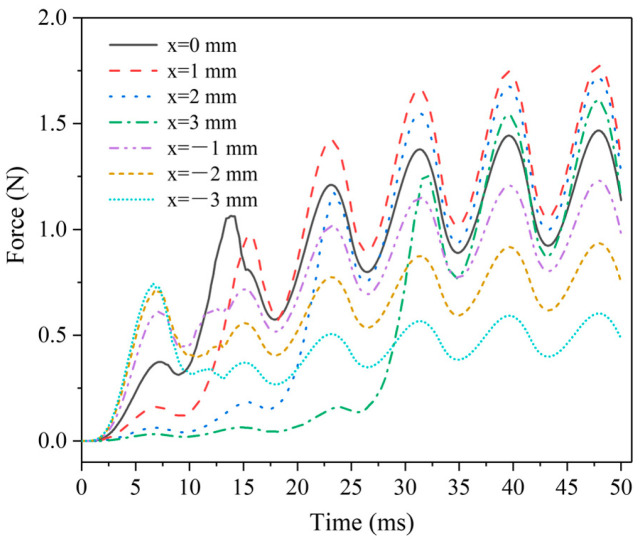
Electromagnetic force dynamic change curves at different positions of MIR.

Figure 5 shows the electromagnetic force dynamic change curves of the AC solenoid valve at different MIR positions. The fluctuation of the electromagnetic force comes from the excitation of the AC power supply. The electromagnetic force’s magnitude and trend remain stable after 25 ms. The changing trend of the solenoid valve is essentially caused by the regulation of the magnetic circuit in the air gap by the spacer ring. When the MIR position *x_z_* < 0 mm, a large electromagnetic force response is obtained in the initial stage, but the average electromagnetic force is low. At *x_z_* = −3 mm, the subsequent electromagnetic force is even significantly lower than the initial response electromagnetic force. Conversely, the initial response electromagnetic force becomes weaker and weaker as *x_z_* increases positively. Especially at *x_z_* = 3 mm, the electromagnetic force basically has no obvious response in the first 25 ms. It is obvious that *x_z_* has better performance in the (−1 mm, 1 mm) interval, the electromagnetic force response is faster, and the value of the force is also larger. 

**Figure 6:**
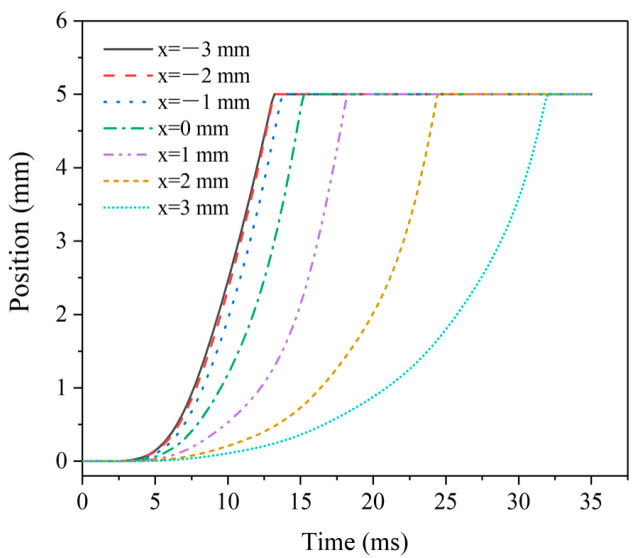
Dynamic displacement curves of MIR at different positions.

Figure 6 shows the dynamic displacement curves of moving iron core at different MIR positions. When the MIR position *x_z_* < 0 mm, the moving iron core displacement curve has little change, and the response performance is better. The response dynamic process and time are basically unchanged, and the experimental results can also be confirmed from the data in Table 2. Subsequently, with the increase of *x_z_*, the time required for displacement becomes larger and larger, and the moving speed of the moving iron core becomes more slowly. Even at *x_z_* = −3 mm, the time required for displacement reaches 32 s, and the displacement time is higher than two times the shortest. The electromagnetic force mainly limits the displacement time on the moving iron core.

### 4.4. Effect of MIR on Magnetic Field Intensity and Distribution of Magnetic Force Lines

**Figure 7:**
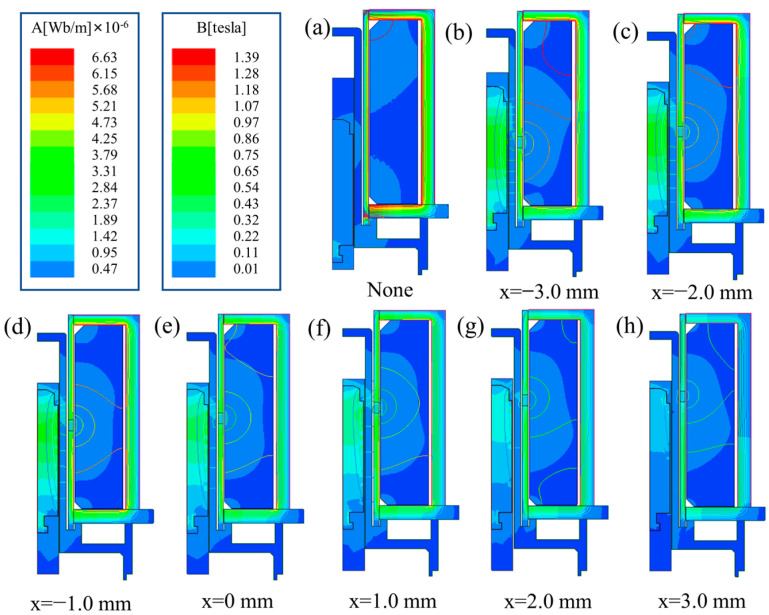
The distribution of the magnetic induction intensity at (**a**) without MIR and MIR’s position at (**b**) −3 mm, (**c**) −2 mm, (**d**) −1 mm, (**e**) 0 mm, (**f**) 1 mm, (**g**) 2 mm, (**h**) 3 mm.

In order to explore the effect of MIR’s position on electromagnetic force and response time, the size and distribution of the magnetic induction intensity of this model were studied. The simulation results are shown in Figure 7. There is no doubt that the position of the MIR has a significant effect on the magnetic field distribution inside the solenoid valve.

As the position of the MIR moves from bottom to top, the maximum magnetic induction intensity area gradually changes from the upper section of the magnetic guide sleeve to the lower end, and the peak value of the magnetic induction intensity generally shows a downward trend. The change of the MIR’s position can affect the change of the magnetic induction intensity in the valve body. It affects the response time of the solenoid valve, and the electromagnetic force after the moving iron core is attracted.

During the design process, the solenoid force of the solenoid valve is calculated according to the following Formula (9):

(9) $$F=\frac{1}{2\mu_{0}}B_{0}^{2}S_{0}\times 10^{6}$$

where *F* is the electromagnetic force, *μ*_0_ is the air permeability, *B*_0_ is the magnetic field intensity in the air gap, and *S*_0_ is the gap area. It is evident that under the structure of the solenoid valve is unchanged, the size of the magnetic force is proportional to the square of the magnetic induction strength in the air gap. The role of the MIR is mainly manifested in the change of the magnetic path.

As shown in Figure 7a, in the absence of the MIR, the magnetic field forms a loop in the conductive sleeve and cannot produce an excellent magnetic field effect in the air gap. As shown in Figure 7b–h, the magnetic line near the MIR is the strongest. The magnetic field strength is higher where the magnetic lines are dense. With the positive movement of the position of the MIR, the dense area of magnetic field lines gradually approaches the air gap position. The strength of the magnetic field in the air gap increases, and the electromagnetic force on the moving iron core also increases. However, when the moving iron core exceeds a specific limit, the effect on the moving iron core is weakened to a certain extent, and the strength of the air gap magnetic field is relatively weakened. Therefore, it can be obtained from the simulation results that MIR’s position is affected by changing the magnetic circuit to affect the dynamic response characteristics of the solenoid valves.

### 4.5. Optimized Design for Dynamic Response Performances

**Figure 8:**
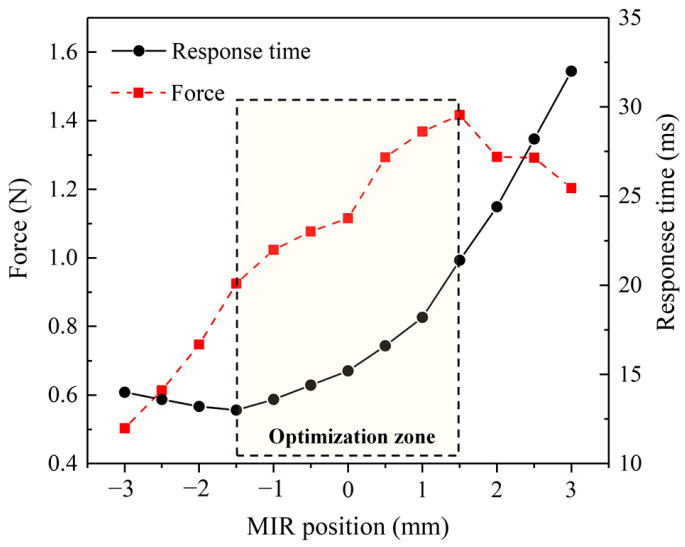
Variation curve of electromagnetic force and response time with MIR.

As shown in Figure 8, the electromagnetic force is the largest when *x_z_* is 1.5 mm. However, the optimal response time value occurs when *x_z_* is −1.5 mm. When *x_z_* is greater than this position, the response time gradually increases, and the rate increases continuously. Finally, the two gradually tend to be linear. Through analysis, a reasonable selection interval is obtained. This position interval is (−1.5 mm, 1.5 mm). Within this interval, the two performance parameters have the same trend with the increase of MIR position. The ability of the solenoid valve to respond quickly to overcome the electromagnetic force is worse when the electromagnetic force increases.

The position of the MIR can affect the magnitude of the electromagnetic force and the response time of the armature at the same time. The effects on the two aspects are conflicting. In general, too high an electromagnetic force can seriously inhibit the optimization of response time. It is always expected to obtain a larger electromagnetic force and a faster response time in practical production applications. A reasonable trade-off between the two is required when the two cannot be satisfied simultaneously. In the case where the application environment of the solenoid valve requires a faster response time, and the electromagnetic force is not strictly required, the optimization design parameter is where the MIR position *x_z_* is −1.5 mm. The response time can reach 13 ms. In contrast, the maximum electromagnetic force can be obtained at the MIR position of 1.5 mm; i.e., the electromagnetic force can be greater than 1.4 N. This method can provide certain theoretical guiding significance for the optimization of the solenoid valves.

## 5. Conclusions

In this paper, the optimization design of MIR position in AC solenoid valves for the dynamic response performance based on a performance-oriented design idea is emphasized. A model of an AC solenoid valve considering the MIR is proposed and established. A series of innovative measurement platforms were developed to detect dynamic response characteristics. The electromagnetic force, response time, and magnetic field strength of the AC solenoid valve are measured by using the developed platform. The influence law of the dynamic response characteristics of the AC solenoid valve is explored and clarified. Finally, the performance-oriented solenoid valve optimization design is realized. The results obtained are summarized as follows:

(1) Through the experimental verification of the developed detection platform, the error rate of the proposed AC solenoid valve simulation model is generally stable at about 10%, which meets the accuracy requirements of the solenoid valve structure optimization.
(2) The electromagnetic force affected after armature absorption increases first and then decreases with the increase of *x_z_* position and reaches the maximum value at *x_z_* = 1.5 mm and *F_max_* = 1.416 N. Response time decreased first and then increased and reached a minimum at *x_z_* = −1.5 mm, with *t_min_* = 13.0 ms.
(3) The position of the magnetic partition ring changes the distribution of the magnetic path in the solenoid valve and then changes the magnetic flux in the air gap, thus affecting the dynamic response characteristics.
(4) The solenoid valve used in this study is an example, the optimal interval of the magnetic ring position is (−1.5 mm, +1.5 mm), in which the solenoid valve has a relatively balanced dynamic response characteristic.

In the subsequent research work, based on the proposed solenoid valve simulation model, the multi-objective parameter optimization of the solenoid valve combined with intelligent algorithms will better realize the high-performance design and manufacture.

## Figures and Tables

**Table 1:** Part material setting.

Item	Setting
Moving iron core	Steel-1008
Magnetic isolation tube	Polyester
Coil	Copper
Magnetic isolation ring	Copper
Concentrating flux sleeve	Iron
Concentrating flux plate	Steel-1008
Solution region	Vacuum
Motion domain	Vacuum

**Table 2:** Electromagnetic force and response time at different MIR positions.

*x_z_*/mm	*t*/ms	*f*/N
−3.0	14.0	0.503
−2.5	13.6	0.614
−2.0	13.2	0.747
−1.5	13.0	0.925
−1.0	13.6	1.023
−0.5	14.4	1.077
0	15.2	1.115
0.5	16.6	1.293
1.0	18.2	1.368
1.5	21.4	1.416
2.0	24.4	1.294
2.5	28.2	1.292
3.0	32.0	1.203

## References

[1] Murakami T., Kuwajima Y., Wiranata A., Minaminosono A., Shigemune H., Mao Z.-B., Maeda S. (2021). A DIY fabrication approach for ultra-thin focus-tunable liquid lens using electrohydrodynamic pump. Micromachines.

[2] Bammesberger S.B., Kartmann S., Tanguy L., Liang D., Mutschler K., Ernst A., Zengerle R., Koltay P. (2013). A low-cost, normally closed, solenoid valve for non-contact dispensing in the sub-µl range. Micromachines.

[3] Kartmann S., Koltay P., Zengerle R., Ernst A. (2015). A disposable dispensing valve for non-contact microliter applications in a 96-well plate format. Micromachines.

[4] Zhang Q., Zhang P.-R., Su Y.-T., Mou C.-B., Zhou T., Yang M.-L., Xu J., Ma B. (2014). On-demand control of microfluidic flow via capillary-tuned solenoid microvalve suction. Lab. Chip..

[5] Yang M.-S. (2022). Study on dynamic and static performance of a micro digital hydraulic valve. Micromachines.

[6] Wang S.-M., Miyano T., Hubbard M. (1993). Electromagnetic field analysis and dynamic simulation of a two-valve solenoid actuator. IEEE Trans. Magn..

[7] Cai B.-P., Liu Y.-H., Tian X.-J., Wang Z.-L., Wang F., Li H., Ji R.-J. (2011). Optimization of Submersible Solenoid Valves for Subsea Blowout Preventers. IEEE Trans. Magn..

[8] Liu Q.-F., Bo H.-L., Qin B.-K. (2013). Optimization of direct-action solenoid valve based on CloudPSO. Ann. Nucl. Energy..

[9] Diao K., Sun X., Lei G., Guo Y., Zhu J. (2020). Multi-objective system level optimization method for switched reluctance motor drive systems using finite-element model. IEEE Trans. Ind. Electron..

[10] Santos G.D., Sass F., Sotelo G.G., Fajoni F., Baldan C.A., Ruppert E. (2021). Multi-objective optimization for the superconducting bias coil of a saturated iron core fault current limiter using the t-a formulation. Supercond. Sci. Technol..

[11] Xu B., Shen J., Liu S.-H., Su Q., Zhang J.-H. (2020). Research and development of electro-hydraulic control valves oriented to Industry 4.0: A review. Chin. J. Mech. Eng..

[12] Shukla V., Vaghela H., Madeenavalli S., Dash B.R., Garg A. (2020). Effect of magnetic field environment on the performance of 3/2 solenoid valve. Fusion. Eng. Des..

[13] Subic A., Cvetkovic D. (2008). Virtual design and development of compact fast-acting fuel injector solenoid actuator. Int. J. Veh. Des..

[14] Hung N.B., Lim O., Yoon S. (2017). Effects of Structural Parameters on Operating Characteristics of a Solenoid Injector. Energy Procedia.

[15] Grekhov L., Zhao J., Ma X. Fast-Response solenoid actuator computational dimulation for engine fuel systems. Proceedings of the 2017 International Conference on Industrial Engineering, Applications and Manufacturing (ICIEAM).

[16] Liu P., Fan L.-Y., Hayat Q., Xu D., Ma X.-Z., Song E.-Z. (2014). Research on key factors and their interaction effects of electromagnetic force of high-speed solenoid valve. Sci. World J..

[17] Hung N.B., Lim O. (2019). Improvement of electromagnetic force and dynamic response of a solenoid injector based on the effects of key parameters. Int. J. Automot. Technol..

[18] Yang M., Zhang J., Xu B. (2018). Experimental study and simulation analysis on electromagnetic characteristics and dynamic response of a new miniature digital valve. Adv. Mater. Sci. Eng..

[19] Zhao J., Yue P., Wei K. (2020). Eddy current effects on the dynamic response of high-speed solenoid valve for common rail injector. Int. J. Appl. Electromagn. Mech..

[20] Liao Y.-Y., Lian Z.-S., Yuan H.-B., Feng J.-L., Cui H.-W. (2019). Optimization of an intrinsically safe solenoid valve and the static and dynamic characteristics. Int. J. Appl. Electromagn. Mech..

[21] Guo D.-M. (2018). High-performance precision manufacturing. China Mech. Eng..

[22] Cao J.-N., Liu J., Yang Y. (2012). Impact characteristics study on electromagnet magnetic isolation ring to electromagnetic force. Appl. Mech. Mater..

[23] Zhang X., Lu Y., Li Y., Zhang C., Wang R. (2019). Numerical calculation and experimental study on response characteristics of pneumatic solenoid valves. Meas. Control.

